# Comparing the effectiveness of game literacy education and game coding education in improving problematic internet gaming

**DOI:** 10.3389/fpsyt.2024.1377231

**Published:** 2024-03-22

**Authors:** Ellie Seunga Han, YeJi Park, Deborah Yurgelun-Todd, Perry F. Renshaw, Doug Hyun Han

**Affiliations:** ^1^ Department of Political Science, Vanderbilt University, Nashville, TN, United States; ^2^ IT and Human Research and Clinic Center, Chung Ang University Medical School, Seoul, Republic of Korea; ^3^ Huntsman Mental Health Institute, University of Utah, Salt Lake City, UT, United States; ^4^ Veterans Affairs (VA) Salt Lake City Mental Illness Research, Education, and Clinical Centers (MIRECC), Salt Lake City, UT, United States; ^5^ Department of Psychiatry, Chung Ang University Hospital, Seoul, Republic of Korea

**Keywords:** internet gaming disorder, game coding education, self-esteem, depressive symptoms, game literacy

## Abstract

**Objective:**

Problematic internet gaming by adolescents has been thought to be associated with low self-esteem, depression, anxiety, and attention problems. We hypothesized that both game literacy and coding education would effectively improve problematic internet use. However, game coding education would be more effective in enhancing self-esteem and social anxiety in adolescents than game literacy education.

**Methods:**

A total of 733 adolescent volunteers were included and randomly assigned to either the game coding education or game literacy education programs. Both programs consisted of eight sessions, each lasting 45 minutes, over four weeks. The coding education sessions included game planning and development lessons and allowed students to create the game’s characters, stages, and tutorials directly using Scratch, a free coding program. Game literacy education sessions included lessons on enjoying gaming with a healthy rationale and etiquette. Data on demographics, gaming patterns, and psychological status, including positive/negative perceptions of online games, depression, social anxiety, and self-esteem, were collected.

**Results:**

Both game coding and game literacy education significantly improved YIAS scores compared to baseline, and there was no significant difference in the YIAS scores between the two groups after the interventions. In the hierarchical logistic regression analysis of all participants, higher YIAS scores, stronger negative perceptions of gaming, and lower attention problem scores at baseline predicted lower levels of internet gaming addiction after interventions. In the hierarchical logistic regression analysis among individuals with game coding education, higher YIAS scores, stronger negative perceptions of gaming, lower attention problem scores, and higher self-esteem scores at baseline predicted lower levels of internet gaming addiction after intervention. In addition, game coding education greatly improved negative perceptions of games, self-esteem, and social anxiety compared to game literacy education.

**Conclusion:**

Both game literacy and game coding education effectively mitigate internet game addiction. However, game coding education effectively mitigated problematic internet gaming by improving negative perceptions of games, self-esteem, and social anxiety in adolescents. We found that the application of knowledge by students in creating their own games was more effective than simply developing a conceptual understanding of the games.

## 1 Introduction

### 1.1 Problematic internet use in children and adolescents

The popularity of internet gaming has increased dramatically in recent years due to the allure of expansive virtual realms and opportunities to control virtual alter egos (1, 2). While participation in internet gaming has increased across all ages, with many adolescents engaging in healthy internet gaming, some gamers are at a heightened risk of developing an Internet gaming disorder (IGD) (2), which is a condition characterized by persistent and recurrent engagement in internet gaming activities to the extent that it leads to significant impairment or distress (3). Notably, IGDs are highly prevalent in Asia (9.9%) and North America (9.4%) compared to other parts of the world (1). Furthermore, in 2020, at the height of the COVID-19 pandemic, the Korea Creative Content Agency’s Gamer Report unveiled that 91.5% of South Korean teenagers actively engaged in online gaming (4). The alarmingly increasing frequency of IGD has led to its recognition as a mental health disorder (5) and a major public health problem (6).

### 1.2 Self-efficacy and anxiety problems associated with IGD

The impact of early exposure to the Internet during childhood has been associated with lower cognitive abilities and academic performance in later years, as children mature into adolescence (7). Indeed, Young et al. further proposed that poor academic performance impacts the self-esteem of individuals and encourages online gaming as a coping mechanism for negative self-perceptions (8). A study by Beard et al. further substantiated this theory by establishing a relationship between early initiation of online gaming, IGD, and self-esteem (9). Their findings indicated that earlier initiation of internet gaming during childhood and adolescence introduced gaming-contingent self-worth as a mediating factor between early initiation and IGD. This suggests that individuals exposed to online gaming early in life rely on these games to regulate their sense of self-worth.

Individuals with gaming-contingent self-worth also exhibited lower global self-esteem, portraying less self-efficacy or individual perception of control over life events in their everyday experiences. Therefore, individuals exposed to internet gaming earlier in life face a greater risk of problematic use owing to their dependence on gaming for an internalized feeling of self-worth compared to those who start online gaming at a later age. In addition, Young et al. proposed that decreased confidence in real-life communication among online game users, coupled with an increased sense of comfort in online communication, may also contribute to poor self-esteem and feelings of isolation in adolescent gamers (8). The estimated decrease in self-esteem due to online gaming across numerous studies is particularly alarming since adolescence is a pivotal period for identity development and the cultivation of real-world self-efficacy (9). Furthermore, low self-esteem in adolescents is linked to increased anxiety, depression, and suicidal ideation. The long-term effects of low self-esteem underscore the imperative need for targeted interventions when treating IGD in adolescents.

### 1.3 Treatment of problematic internet gaming through game literacy education and game coding education

The recent inclusion of a unified definition of IGD in the DSM-5 has spurred a notable increase in research on this disorder. There were various approaches, including cognitive behavior therapy, medications, literacy education, and coding education for treating problematic internet use or IGD (10–17). Compared to traditional treatments, including cognitive behavior therapy and medications, game literacy and coding education were considered effective for preventing problematic internet use in large groups of participants by understanding, approaching, interpreting, and evaluating games by considering human factors (10).

Several studies have applied game literacy and coding education to control internet use patterns (11–13). Jiang et al. (11) reported that internet literacy education, including knowledge and skills for internet self-management, could prevent the risk of problematic internet use. Liu et al. (12) also declared that internet literacy education prevented problematic internet use in adolescents. Jeon et al. (13) provided empirical evidence that mental health literacy of IGD would prevent the prevalence of IGD in Korean teenagers.

Game literacy demonstrates a player’s ability to effectively use game-related information, including understanding, approaching, interpreting, and evaluating games by considering human factors (10). A player with high game literacy knows how to consume and follow the formal and informal gameplay rules (14). However, the application of game literacy education to adolescents is constrained due to its demands for prolonged attention and a willingness to learn.

Game coding education is one possible treatment that addresses self-esteem in adolescents with problematic internet gaming behavior (15–17). Many countries globally are initiating coding programs, recognizing their potential to instill diverse skills beyond coding, such as problem-solving, computational thinking, persistence, confidence, and communication skills (15). In the study conducted by Soyokan and Kanbul involving 6th and 7th-grade students, the application of coding education resulted in elevated levels of self-efficacy compared to those who did not receive coding education (16). Furthermore, in a preliminary iteration of this study, Chung et al. demonstrated that implementing game coding education substantially reduced problematic internet gaming and increased self-esteem (17). Therefore, treatment approaches should prioritize effective problem-solving and the social skills required to improve self-efficacy and self-esteem in adolescents with problematic internet gaming behaviors. However, the application of game coding education to adolescents is constrained due to its demands for prolonged attention and a willingness to learn. Moreover, the number of studies on coding education was fewer than that on game literacy.

### 1.4 Hypotheses

We hypothesized that game literacy and game coding education programs would reduce problematic internet gaming. However, compared to game literacy education, game coding education fostering problem-solving skills, logical reasoning, and computational thinking would be more effective in improving self-esteem. Further, we predicted that reduced problematic internet gaming and improved self-esteem would be associated with improvements in depression, anxiety, and attention problems.

## 2 Methods

### 2.1 Participants recruitment

A sample size of 779 was used in the current study to calculate the G-power (effect size = 0.1, α = 0.05, and power = 0.8) (18). Considering the 30% dropout rate, we planned to recruit 1100 students for the current study. The inclusion criteria were as follows: 1) students aged from 10 to 15 in elementary or middle schools and 2) students who can understand the teacher’s instruction. The exclusion criteria were as follows: 1) low intelligence students who could not understand the teacher’s instruction and could not answer in response to demographic and clinical scales. The dropout criteria were as follows: 1) students who missed more than three sessions of game literacy or game coding education and 2) students who missed data storage in baseline or follow-up surveys.

Over three years, 733 children and adolescents from 58 elementary schools and 57 middle schools across 23 regions in South Korea actively engaged in a “Visiting Game Class” program after discovering it through an online advertisement on the Korean Game Culture Foundation website (http://www.gameculture.or.kr/).

In the initial phase, 1102 students applied to the Visiting Game Class program. These students were randomized into a game literacy or game coding group. The randomization was performed using Friedman’s Urn model as an adaptive biased coin randomization (19). The Game Culture Foundation generated 1102 slots for education methods (551 game literacy and 551 game coding). When the school applied for education and notified the list, the Game Culture Foundation informed them of the education method (game literacy or game coding) according to the order of application.

**Figure 1:**
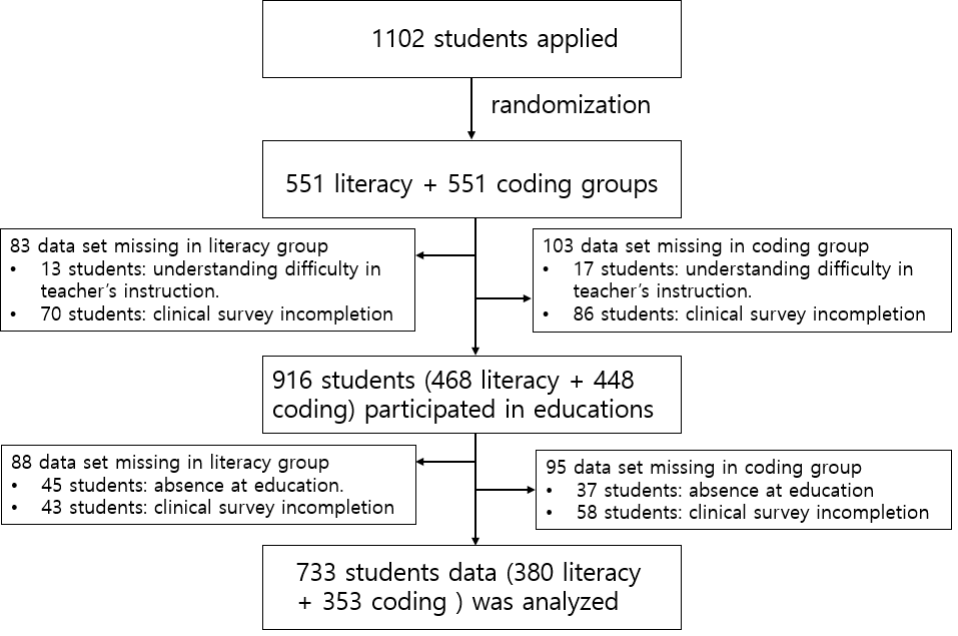
STROBE flow chart of participant enrollment.

Of these, 186 students (83 in the game literacy group and 103 in the game coding group) were ruled out. A total of 13 students in the literacy group and 17 in the game coding group could not understand the teacher’s instructions. Seventy students in the game literacy group and eighty-six in the game coding group did not complete the clinical survey at baseline. At the end of the research, 183 students (83 in the game literacy group and 95 in the game coding group) were excluded. A total of 45 students in the literacy group and 37 in the game coding group were absent from education more than three times. A total of 43 students in the literacy group and 58 in the game coding group did not complete the clinical survey at the end of the research. Even incomplete, missing data from a single questionnaire were not utilized in the analysis of this study. Consequently, data analysis was conducted based on the information from 733 students, with 380 in the literacy group and 353 in the coding education group (**Figure 1**).

While there was no monetary compensation for participation, participants were awarded free game literacy or coding education. Approval for this study was granted by the Institutional Review Board at Chung-Ang University (1041078-202201-HR-052).

### 2.2 Procedures and assessment

#### 2.2.1 Game literacy education and game coding education

The Game Culture Foundation trained 20 professional educators in both game literacy (n = 10) and coding (n = 10) for 6 months. An experienced educator visited each school for game literacy or coding education. The education sessions were conducted in each school after regular classes. The homeroom teachers encouraged students to participate in education actively. The game literacy and coding education programs were created through open calls. The education duration and content were determined through discussions among the groups responsible for each production, with sessions set at durations of 45–50 minutes each and a total of eight sessions.

Participants were randomly allocated to a game coding or game literacy education program. The game literacy education sessions centered on understanding gameplay rationale, cultivating an appreciation for games, and instilling rules and etiquette for engaging in gaming activities. Through this program, participants actively exchanged opinions and perceptions on the specific types of games they commonly played. The contents of literacy education were as follows: self-introduction through the game (session 1), how to enjoy gaming wisely (session 2), reflecting on why they gamed (session 3), identifying obstacles that hinder gaming (session 4), discussion about gameplay with other participants (session 5), how to game safely (lesson 6), discussion about jobs related to gaming (session 7), and creating personal game stories (session 8).

The coding education sessions focused on instructing students in the game planning and development processes, enabling them to actively create game characters, stages, and tutorials using Scratch, a freely available coding platform. While some content variations were based on grade level, all coding education sessions involved the fundamental aspects of game coding. The contents of coding education were as follows: moving game characters (session 1), creating obstacles and game reset functions (session 2), creating the “blue flag, white flag” game (session 3), checking correct answers, and creating initialization functions (session 4), coding to move characters and obstacles at the same time (session 5), creating setting window and touch effects (session 6), saving the score to the database and unlocking characters (session 7), and signing and sharing with friends (session 8).

#### 2.2.2 Demographics and internet use patterns

Based on clinical and psychological scales adopted from past studies on internet usage or internet gaming (17, 20), questionnaires evaluating internet usage patterns and clinical scales, including psychological state and underlying diseases, were selected.

Demographic data included age, gender, and school year. Additionally, participants completed items assessing their internet use pattern, including the extent of internet addiction levels and individuals’ attitudes towards internet games.

The Young’s Internet Addiction Scale (YIAS) is a widely utilized tool for evaluating internet addiction or any online activities, including gaming. It comprises 20 items, each rated on a 5-point Likert scale (20). The internal consistency of the Korean version of the scale (K-YIAS) has been documented to fall within the range of 0.90 to 0.93 (21).

The Internet Game Literacy Scale (IGLS) assesses individuals’ attitudes toward Internet games, gauging whether their perception is positive or negative. This scale consists of nine items, each rated on a 5-point Likert scale, ranging from 1 (“strongly disagree”) to 5 (“strongly agree”). The internal consistency of the IGLS has been reported as α = 0.89 (22).

#### 2.2.3 Psychological assessment scales

The Patient Health Questionnaire 9 (PHQ-9) was employed to evaluate depression, with each item being rated on a Likert scale from 0 to 3. A depression cutoff point of 10 (out of 27) was utilized (23). The Korean version of the PHQ-9 was validated by Park et al. (2010), revealing an internal consistency of α = 0.81 (24).

The Dupaul ADHD scale, specifically the ADHD symptom severity scale (ARS), consists of 18 items, with 9 items focusing on inattention and 9 on hyperactivity (25). So et al. (2002) validated the Korean version of the ARS scale (K-ARS), reporting an internal consistency within the range of 0.77 to 0.89 (26).

The Social Phobia Inventory (SPIN) is a questionnaire consisting of 17 self-report items designed to assess three dimensions of social anxiety (27). Cho et al. (2018) developed a Korean version of the inventory, referred to as the K-SPIN, and reported a high internal consistency with a Cronbach’s alpha of 0.91 (28).

The Two-Factor Self-Esteem Scale is derived from a modified version of the Rosenberg Self-Esteem Scale, conceptualizing self-esteem as an individual’s perception of their worth, incorporating elements of self-respect and self-confidence (29). This scale comprises 10 statements that evaluate overall sentiments toward oneself. Participants indicate their level of agreement on a 4-point Likert scale, ranging from 1 (“agree not at all”) to 4 (“agree completely”). The internal consistency of the Korean scale version, hereafter referred to as SE Scale-Korean, has been documented as α = 0.79 (30).

### 2.3 Data analysis

Participants were categorized into two distinct groups: (1) those experiencing problematic internet gaming and (2) those engaging in non-problematic internet gaming. The first group comprised individuals with scores on the YIAS equal to or exceeding 40 (31, 32).

We conducted a comparative analysis between the participants enrolled in the two programs: Gaming coding and game literacy. Gender, age, education year, internet use time, and internet activity were examined using chi-square and independent samples t-tests. Additionally, differences across conditions in psychological variables, specifically YIAS, IGLS, PHQ-9, K-ARS, K-SPIN, and self-esteem Scale-Korean, were evaluated using independent samples t-tests.

We conducted hierarchical logistic regression analyses to assess the impact of the variables of interest—the effectiveness of game literacy education vs. game coding education—in the entire sample. The effectiveness of either education session on internet gameplay was operationalized as achieving a YIAS score of less than 40 after education.

Hierarchical logistic regression was thought to account for complex relationships between predictor variables and the outcome variable while also considering group-level confounders. This can be particularly useful when multiple levels of predictors and group levels of predictors influence the outcome. In the current study, the effects of education may be affected by complex relationships between groups (demographic, education type, internet use pattern, and psychological status) and multiple individual-level predictors. In the current hierarchical logistic regression analyses, those individual and group-level confounders were controlled as covariates (33).

As independent variables, we introduced a discrete set of hierarchical variables: Model 1 included demographic factors; Model 2 comprised demographic factors along with the type of gaming education program (game coding education vs. game literacy education); Model 3 incorporated demographic factors, gaming education program type, and internet use pattern; Model 4 involved demographic factors, gaming education program type, internet use, and psychological status.

Additionally, within each education group, hierarchical logistic regression analyses were conducted using the set of variables, including Model 1, 2, and 3, to investigate their effects on the effectiveness of education. As independent variables, we introduced a discrete set of hierarchical variables: Model 1 included demographic factors, Model 2 incorporated demographic factors and internet use patterns, and Model 3 involved demographic factors, internet use patterns, and psychological status.

Lastly, a repeated-measures ANOVA was employed to examine distinctions between the game coding group and the game literacy group regarding alterations in (1) patterns of internet use and (2) psychological status.

## 3 Results

### 3.1 Comparisons of demographic, internet use pattern, and psychological status between groups

There were no differences in age (literacy: 12.6 ± 1.4, coding: 12.5 ± 1.5 years old), gender (literacy: 52.1% (boys) and 47.9% (girls), coding: 51.6% (boys) and 48.4% (girls)), and education year (literacy: 6.6 ± 1.5, coding: 6.5 ± 1.5 years), between the two groups (**Table 1**).

**Table 1:** Demographic, Internet use patterns, and psychological scale in all participants.

	Game literacy group (n = 380)	Game coding group (n = 353)	Statistics
Gender (boys/girls)	198 (52.1%)/182 (47.9%)	182 (51.6%)/171(48.4%)	χ2 = 0.02, p = 0.88
Age (years)	12.6 ± 1.4	12.5 ± 1.5	t = 0.93, p = 0.35
Education (grades)	6.6 ± 1.5	6.5 ± 1.5	t = 0.94, p = 0.35
Internet use time (hours/day)	3.4 ± 1.6	3.3 ± 1.4	t = 0.33, p = 0.74
Internet activity			
Game	127 (33.4%)	123 (34.8%)	χ2 = 8.69, p = 0.08
OTT	154 (40.5%)	158 (44.8%)	
Study	5 (1.3%)	11 (3.1%)	
SNS	91 (23.9%)	58 (16.4%)	
Searching	3 (0.8%)	3 (0.8%)	
YIAS	36.8 ± 8.4	37.2 ± 7.9	t = -0.77, p = 0.44
IGLS-pos	30.0 ± 6.5	29.1 ± 7.2	t = 1.80, p = 0.07
IGLS-neg	22.9 ± 7.4	23.9 ± 7.6	t = -1.80, p = 0.07
PHQ-9	12.3 ± 4.8	12.8 ± 5.0	t = -1.60, p = 0.11
K-ARS	7.9 ± 8.2	8.1 ± 7.4	t = -0.34, p = 0.73
KSPIN	19.2 ± 13.0	19.4 ± 12.8	t = -0.19, p = 0.85
Self-esteem	26.5 ± 4.9	26.5 ± 5.5	t = -0.24, p = 0.98

YIAS, Young’s Internet Addiction Scale; OTT, over-the-top; SNS, social network service; IGLS Pos/Neg, Internet Game Literacy Scale Positive/Negative; KSPIN, Korean-Social Phobia Inventory; PHQ-9, Patient Health Questionnaire-9; K-ARS, Korean Attention Deficit Hyperactivity Disorder Scale.

There were no differences in internet use time (literacy: 3.4 ± 1.6 hours/day, coding: 3.3 ± 1.4 hours/day), YIAS scores (literacy: 36.8 ± 8.4, coding: 37.2 ± 7.9), IGLS positive (literacy: 30.0 ± 6.5, coding: 29.1 ± 7.2), negative scores (literacy: 22.9 ± 74, coding: 23.9 ± 7.6), and internet activity between the two groups. In both groups, gaming (literacy: 33.4%, coding: 34.8%) and over-the-top (OTT) streaming services (literacy: 40.5%, coding: 44.8%) were the most frequently used modes of internet activities.

There was no significant difference in the number of participants exhibiting problematic internet gameplay between the game literacy group (*n* = 92; 24.2%) and the game coding group (*n* = 96; 27.2%; χ^2 ^= 0.86, *p* = 0.39)

A total of 423 (90.3%) students in the literacy group and 411 (91.7%) students in the game coding group completed their educations.

### 3.2 Hierarchical logistic regression for effectiveness of education

Two of the four models tested in all participants yielded results attesting to the effectiveness of all education (literacy and coding) in the total sample. Model 3 yielded χ^2 ^= 93.610 (*p* < 0.001) and Nagelkerke’s *R* ^2 ^= 0.259 (25.9% of the variance in the dependent variable explained), indicating that the model was adequate for predicting the effect of the educational programs. With stepwise χ^2 ^= 93.190 (*p* < 0.001), the internet use pattern was a significant predictor of the effectiveness of all education programs. Model 4 yielded χ^2 ^= 109.832 (*p* < 0.001) and Nagelkerke’s *R* ^2 ^= 0.301 (30.1% of the variance in the dependent variable explained), indicating that the model was adequate for predicting the effectiveness of the education programs. With stepwise χ^2 ^= 16.222 (*p* = 0.003), psychological status was a significant predictor. Based on the Wald statistics for all independent variables, YIAS score, stronger negative perceptions of gaming, and K-ARS scores significantly predicted the effectiveness of game education (literacy or coding) (**Table 2**).

**Table 2:** Hierarchical regression analysis for effectiveness in game literacy and game coding education.

Independent variables		Model 1			Model 2			Model 3			Model 4		
		B	Wald	OR	B	Wald	OR	B	Wald	OR	B	Wald	OR
Demographics	Gender	0.134	0.276	1.143	0.136	0.283	1.146	0.200	0.405	1.222	0.146	0.195	1.157
	Age	0.130	0.280	1.133	0.140	0.292	1.152	0.202	0.425	1.119	0.152	0.200	1.162
	Education	0.036	0.164	1.037	0.040	0.165	1.041	0.037	0.091	1.037	0.000	0.000	1.000
Game education	Type				0.024	0.008	1.024	0.034	0.011	1.034	0.061	0.036	1.063
Internet use patterns	Time							0.016	0.028	1.017	0.037	0.136	1.038
	Activity							0.189	1.970	1.208	0.099	0.476	1.104
	YIAS							0.120	58.370	1.128**	0.137	52.317	1.147**
	IGLS-pos							0.011	0.220	1.011	0.016	0.387	1.016
	IGLS-neg							0.039	3.418	1.040	0.051	4.877	1.053*
Psychological status	PHQ-9										0.016	0.178	1.016
	K-ARS										-0.074	11.504	0.928**
	KSPIN										0.010	0.524	1.010
	Self-esteem										1.015	0.245	1.009
Indices	Model 0	Model 1			Model 2			Model 3			Model 4		
-2LL	456.364	455.985			455.978			362.788			346.566		
Step χ^2^/*p*	N/A	0.413/0.814			0.008/0.930			93.190/< 0.001**			16.222/0.003**		
Model χ^2^/*p*	N/A	0.413/0.814			0.420/0.936			93.610/< 0.001**			109.832/< 0.001**		
Nagelkerke’s *R* ^2^	N/A	0.001			0.001			0.259			0.301		
Class accur	90.5	90.5			90.5			90.2			90.5		

* p < 0.05, ** p < 0.01; -2LL, -2 log-likelihood; Class accur, classification accuracy; YIAS, Young’s Internet Addiction Scale; IGLS pos/neg, Internet Game Literacy Scale positive/negative; KSPIN, Korean-Social Phobia Inventory; PHQ-9, Patient Health Questionnaire-9; K-ARS, Korean Attention Deficit Hyperactivity Disorder Scale.

Of the three models tested in each group, two yielded results consistent with the effectiveness of the game literacy education. Model 2 yielded χ^2 ^= 53.688 (*p* < 0.001) and Nagelkerke’s *R* ^2 ^= 0.287 (28.7% of the variance in the dependent variable explained), indicating that the model was adequate for predicting the effectiveness of game literacy education. With stepwise χ^2 ^= 54.110 (*p* < 0.001), the internet use pattern was a significant predictor of the effectiveness of game literacy education. Model 3 yielded χ^2 ^= 60.315 (*p* < 0.001) and Nagelkerke’s *R* ^2 ^= 0.317 (31.7% of the variance in the dependent variable explained), indicating that the model was adequate for predicting the effect of the game literacy education. However, psychological status alone was not a significant predictor (stepwise χ^2 ^= 6.205 (*p* = 0.184)) (**Table 3**).

**Table 3:** Hierarchical regression analysis for effectiveness in game literacy.

Independent variables		Model 1			Model 2			Model 3		
		B	Wald	OR	B	Wald	OR	B	Wald	OR
Demographics	Gender	0.230	0.230	1.258	0.279	0.402	1.322	0.248	0.293	1.282
	Age	0.241	0.258	1.102	0.284	0.411	1.257	0.254	0.303	1.300
	Education	0.015	0.015	1.015	-0.003	0.000	0.997	-0.026	0.027	0.974
Internet use patterns	Time				-0.107	0.587	0.898	-0.068	0.219	0.934
	Activity				0.095	0.267	1.100	0.031	0.025	1.032
	YIAS				0.122	29.056	1.129**	0.123	24.433	1.131**
	IGLS-pos				0.004	0.011	1.004	0.013	0.116	1.013
	IGLS-neg				0.037	1.527	1.038	0.051	2.371	1.052
Psychological status	PHQ-9							0.051	0.970	1.052
	K-ARS							-0.070	4.506	0.933*
	KSPIN							-0.003	0.031	0.997
	Self-esteem							-0.048	1.321	0.953
Indices	Model 0	Model 1			Model 3			Model 4		
-2LL	241.054	236.731			183.043			176.838		
Step χ^2^/*p*	N/A	0.422/0.810			53.688/< 0.001**			6.205/0.184		
Model χ^2^/*p*	N/A	0.422/0.810			54.110/< 0.001**			60.315/< 0.001**		
Nagelkerke’s *R* ^2^	N/A	0.002			0.287			0.317		
Class accur	90.4	90.4			91.2			91.2		

* p < 0.05, ** p < 0.01; -2LL, -2 log-likelihood; Class accur, classification accuracy; YIAS, Young’s Internet Addiction Scale; IGLS pos/neg, Internet Game Literacy Scale positive/negative; KSPIN, Korean-Social Phobia Inventory; PHQ-9, Patient Health Questionnaire-9; K-ARS, Korean Attention Deficit Hyperactivity Disorder Scale.

Of the three models tested in the current study, two yielded results consistent with the effectiveness of the game coding education. Model 2 yielded χ^2 ^= 43.427 (*p* < 0.001) and Nagelkerke’s *R* ^2 ^= 0.250 (25.0% of the variance in the dependent variable explained), indicating that the model was adequate for predicting the effectiveness of the game coding education. With stepwise χ^2 ^= 43.069 (*p* < 0.001), the internet use pattern was a significant predictor of the effectiveness of the game coding education. Model 3 yielded χ^2 ^= 60.485 (*p* <.001) and Nagelkerke’s *R* ^2 ^= 0.317 (31.7% of the variance in the dependent variable explained), indicating that the model was adequate for predicting the effect of the game literacy education. With stepwise χ^2 ^= 17.059 (*p* = 0.01), psychological status was a significant predictor. Based on the Wald statistics for all independent variables, YIAS score, lower K-ARS scores, and higher self-esteem all significantly predicted the effectiveness of game education (**Table 4**).

**Table 4:** Hierarchical regression analysis for effectiveness in game coding education.

Independent variables		Model 1			Model 2			Model 3		
		B	Wald	OR	B	Wald	OR	B	Wald	OR
Demographics	Gender	0.029	0.006	1.029	0.126	0.074	1.135	0.056	0.013	1.057
	Age	0.035	0.004	1.032	0.128	0.075	1.142	0.062	0.014	1.058
	Education	0.107	0.353	1.113	0.185	0.917	1.203	0.084	0.194	1.087
Internet use patterns	Time				0.190	1.645	1.209	0.205	1.801	1.228
	Activity				0.335	2.784	1.398	0.229	1.095	1.258
	YIAS				0.123	29.296	1.131**	0.165	29.441	1.180**
	IGLS-pos				0.026	0.606	1.027	0.024	0.428	1.024
	IGLS-neg				0.043	1.946	1.044	0.057	2.701	1.059
Psychological status	PHQ-9							-0.027	0.229	0.973
	K-ARS							-0.082	6.913	0.922**
	KSPIN							0.027	1.763	1.027
	Self-esteem							0.107	2.027	1.107*
Indices	Model 0	Model 1			Model 2			Model 3		
-2LL	219.061	218.874			175.805			162.746		
Step χ^2^/*p*	N/A	0.357/0.836			43.069/< 0.001**			17.059/0.01*		
Model χ^2^/*p*	N/A	0.357/0.836			43.427/< 0.001**			60.485/< 0.001**		
Nagelkerke’s *R* ^2^	N/A	0.002			0.250			0.317		
Class accur	90.7	90.7			89.2			91.4		

* p < 0.05, ** p < 0.01; -2LL, -2 log-likelihood; Class accur: classification accuracy; YIAS, Young’s Internet Addiction Scale; IGLS pos/neg, Internet Game Literacy Scale positive/negative; KSPIN, Korean-Social Phobia Inventory; PHQ-9, Patient Health Questionnaire-9; K-ARS, Korean Attention Deficit Hyperactivity Disorder Scale.

### 3.3 Comparison of changes in internet use patterns and psychological status between the game literacy and coding education groups

**Figure 2:**
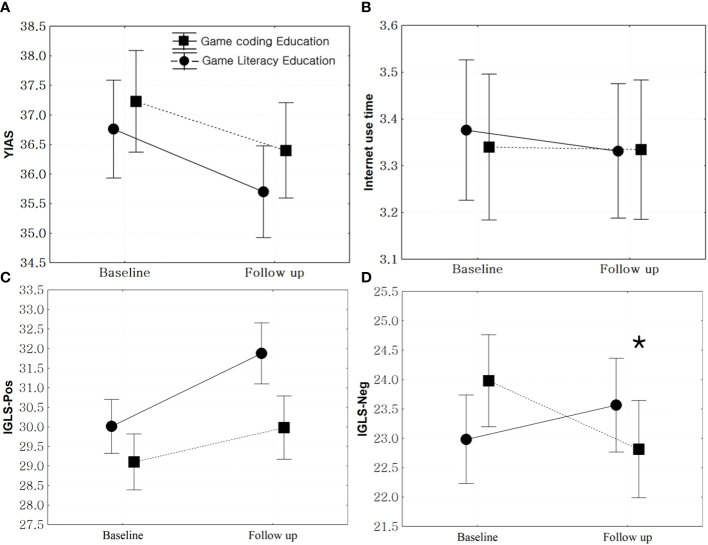
Comparison of the changes in internet use patterns between game literacy and game coding groups. *: statistical significance. **(A)**: Comparison of the changes in Young Internet Addiction Scale (YIAS) scores between game literacy and game coding groups, F = 0.20, p = 0.65. **(B)**: Comparison of the changes in internet use time (hours/day) between game literacy and game coding groups, F = 0.43, p = 0.51. **(C)**: Comparison of the changes in Internet Game Literacy Scale positive perception (IGLS-pos) scores between game literacy and game coding groups, F = 2.90, p = 0.09. **(D)**: Comparison of the changes in Young Internet Addiction Scale Internet Game Literacy Scale negative perception (IGLS-neg) scores between game literacy and game coding groups, F = 7.36, p < 0.01.

Education significantly decreased the YIAS scores in the game literacy (t = 2.81, p = 0.005) and coding education groups (t = 2.39, p = 0.017). However, the two groups had no significant difference in the improved YIAS scores from baseline to follow-up (F = 0.20, p = 0.65). In addition, the internet use time did not change in either the game literacy (t = -0.81, p = 0.11) or the coding education group (t = 0.15, p = 0.88) (**Figure 2**).

IGLS-pos scores increased in the game literacy group (t = -3.70, p < 0.01), while there was no significant change in the coding education group (t = -1.52, p = 0.12). There was no significant difference in the IGLS-pos score changes between the new IGLS-pos scores in the two groups from baseline to follow-up (F = 2.90, p = 0.09). The IGLS-neg scores did not change in the game literacy group (t = -1.02, p = 0.31) but decreased in the coding education group (t = 2.06, p = 0.04). There was a significant difference in the changes in IGLS-neg scores between the two groups (F = 7.36, p < 0.01) (**Figure 2**).

**Figure 3:**
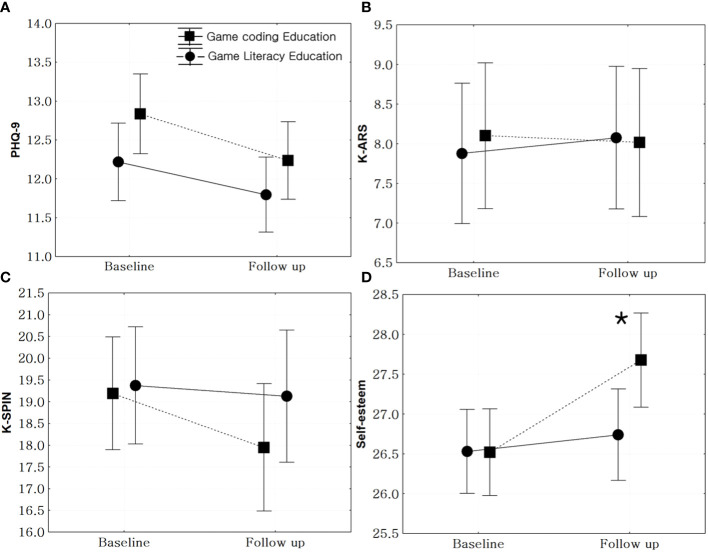
Comparison of the changes in psychological status between game literacy and game coding groups. **(A)**: Patient Health Questionnaire 9 (PHQ-9), F = 0.24, p = 0.62, **(B)**: Dupaul attention deficit hyperactivity disorder (ADHD) scale, F = 0.22, p = 0.64, **(C)**: Korean version of Social Phobia Inventory (K-SPIN), F = 1.03, p = 0.31, **(D)**: Rosenberg Self-Esteem Scale, F = 4.64, p = 0.03, *: statistical significance.

PHQ-9 scores did not decrease in either the game literacy group (t = 1.62, p = 0.11) or the coding education group (t = 1.31, p = 0.19). There was no significant difference in the changes in PHQ-9 scores between the two groups from baseline to follow-up (F = 0.24, p = 0.62). Moreover, the K-ARS scores did not change in either the game literacy (t = -0.31, p = 0.76) or the coding education group (t = 0.13, p = 0.89) (**Figure 3**).

There was no significant change in the K-SPIN scores between the game literacy (t = 0.34, p = 0.73) and coding education groups (t = 1.25, p = 0.21). Additionally, there was no significant difference in the K-SPIN scores between the two groups from baseline to follow-up (F = 1.03, p = 0.31). However, the self-esteem scores significantly increased in the game coding education group, compared to the game literacy education group (F = 4.64, p = 0.03) (**Figure 3**).

**Figure 4:**
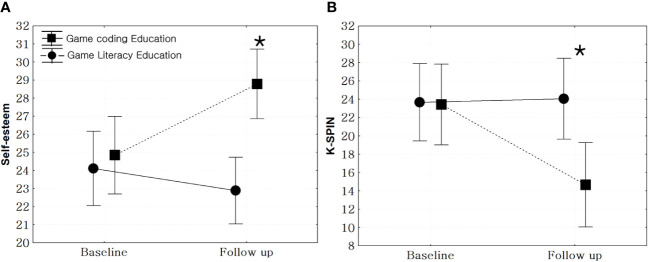
Comparison of the changes in psychological status in the education-effective groups. **(A)**: Rosenberg Self-Esteem Scale, F = 10.61, p < 0.01, **(B)**: Korean version of Social Phobia Inventory (K-SPIN), F = 5.31, p = 0.02, *: statistical significance.

After analyzing the effectiveness of the two education programs, it was noted that the self-esteem scores in the game coding education group significantly increased post-education compared to the game literacy group (F = 10.61, p < 0.01). In addition, the K-SPIN scores in the game coding education group were significantly decreased compared to the game literacy group (F = 5.31, p = 0.02) (**Figure 4**).

## 4 Discussion

In this study, the effectiveness of the game education program in improving problematic internet gaming was predicted by higher YIAS scores, lower K-ARS scores, and higher self-esteem among adolescents with problematic internet gaming behaviors. Additionally, while game literacy and game coding education significantly decreased internet addiction scores, only game coding education lowered negative perceptions of internet gaming. Further, the self-esteem scores in the game coding education group were significantly higher than in the game literacy education group.

### 4.1 Game literacy education

Game literacy education would significantly improve YIAS scores. In addition, game literacy education would also increase positive thinking regarding internet games (not decrease negative thinking concerning internet games). In our literacy education, session 3 of reflecting on why they gamed and session 4 of identifying obstacles that hinder gaming may be associated with increased positive thinking, which would be related to improving YIAS scores. In our literacy education, session 2 of how to enjoy gaming wisely and session 6 of how to game safely may be associated with increased positive thinking. In previous studies, the improvement of YIAS would support the effectiveness of game literacy on IGD (11–13).

Internet use pattern was significantly associated with the effectiveness of both educations. A higher YIAS score could especially predict the effectiveness of education in the current study. However, there was no significant difference in effectiveness between game literacy and game coding education. Considering these results, we can suggest that both educations effectively improve problematic internet use. The reduction in the YIAS score could represent the improvement in problematic internet use patterns (11–13, 21).

### 4.2 Coding education and self-esteem

The rapid increase in interest in coding education can be attributed to its demonstrated impact in developing diverse skills beyond coding, including problem-solving, computational thinking, persistence, confidence, and communication skills (15). In our coding program, session 3, which involved creating a “blue flag and white flag,” and session 4, which involved checking correct answers, were associated with computational thinking. Simultaneously, gaming is also one of the most popular recreational activities for adolescents, serving as a tool to engage students within the educational framework (34) effectively. Game coding education, which allows students to develop and design their own games, can teach students practical skills and increase their self-efficacy and self-esteem (35). In our coding program, session 1 of moving game characters and session 2 of creating obstacles and game reset functions were associated with designing and developing their own games.

Other interventions have been proposed to improve self-esteem in adolescents. Kim et al. found that the reality therapy group counseling program, a widely employed form of therapy for various forms of addiction, resulted in decreased Internet addiction levels and improved self-esteem among South Korean university students with Internet addiction. Reality therapy focuses explicitly on the participant’s direction and self-responsibility in their life choices. Furthermore, it develops the participant’s commitment to making new and difficult choices, naturally improving their self-esteem (36). In addition, Yeun et al. showed that psychosocial intervention, focused on psychological or social factors rather than biological factors, significantly reduced internet addiction and improved self-control and self-esteem among school-aged children with Internet addiction in South Korea (37). In our game coding education program, session 8 of signing and sharing with friends was associated with social factors and communication skills. This educational approach concurrently instilled a sense of achievement and provided healthy coping mechanisms that still retained the elements of gaming adolescents are fascinated with. Therefore, game coding education emerges as a potentially effective but enjoyable treatment method for adolescents with problematic internet gaming behavior.

### 4.3 Coding education, negative perceptions of internet gaming, and depressive symptoms

In this study, stronger negative perceptions of internet gaming predicted improved problematic internet gaming behaviors in all education groups. Moreover, stronger negative perceptions of internet gaming predicted improved problematic internet gaming behaviors in the game coding education group. In addition, the negative perceptions of internet gaming scores were significantly decreased in response to game coding education. In our previous study, a positive perception of internet gaming was associated with protective effects against developing IGD. In contrast, a negative perception of internet gaming was associated with a risk factor for IGD (17). Taken together, we may suggest that game coding education can improve the negative perception of internet games in adolescents with a high negative perception of internet games, which may linked with problematic internet gameplay.

In addition, this phenomenon might be associated with self-efficacy and depressive symptoms. While problematic internet gamers recognized excessive internet gaming as problematic, they continued gaming extensively due to low self-efficacy. For vulnerable adolescents with low self-esteem and self-efficacy, internet gaming may serve as a way of forming an alternate virtual identity that offers players a stronger and more positive sense of self (38). In addition, adolescents with low self-esteem are also at a higher risk of depression during early adulthood (39). Therefore, when analyzing the associated negative impacts of low self-esteem, it becomes imperative for problematic internet gaming treatments to focus on improving their self-esteem.

### 4.4 Limitations

IGD represents a relatively new study area, meaning there is a lack of studies in the current literature. Our study contributes to this evolving field by demonstrating the efficacy of game coding education in reducing problematic internet gaming behavior and various psychological outcomes, including improvements in self-esteem and depressive symptoms. Nevertheless, several limitations should be noted. First, the reliance on questionnaires during data collection lacks a detailed and professional evaluation compared to the participants evaluated by trained experts. In addition, missing data introduces potential biases that may influence the accuracy of our results. Second, in the current study, there was no control group (that did not receive any intervention), which limits the ability to attribute improvements solely to game literacy or coding education. Additionally, the current study’s conclusions may be limited due to the specific cultural and educational context of South Korea. Hence, future studies should include a non-intervention group for more robust comparisons and multicultural backgrounds Third, since the current study could not address the long-term sustainability of education, these remain unclear, given the unequal accessibility to extend coding education opportunities beyond the study. There is a need for longitudinal studies to assess whether the positive effects of game coding education persist over time. Future studies must observe the enduring impacts of future coding education curricula.

## 5 Conclusions

In conclusion, our results demonstrated the efficacy of game coding education, compared to game literacy education, in improving problematic internet gaming behavior in conjunction with participants’ self-esteem. The participants’ engagement in a creative process to construct their achievement proved more effective than developing foundational understandings of the gaming world. In the expanding domain of internet gaming disorder research, our study contributes valuable guidance into potentially effective and engaging treatment modalities for adolescents with problematic internet gaming behavior. Given the increasing accessibility of the Internet to adolescents at younger ages, it becomes imperative for future research to prioritize the development of practical educational curricula that can be implemented in the near future.

## Data availability statement

The original contributions presented in the study are included in the article/supplementary material. Further inquiries can be directed to the corresponding author.

## Ethics statement

The studies involving humans were approved by Institutional Review Board at Chung-Ang University. The studies were conducted in accordance with the local legislation and institutional requirements. Written informed consent for participation in this study was provided by the participants’ legal guardians/next of kin.

## Author contributions

EH: Conceptualization, Data curation, Writing – original draft. YP: Data curation, Writing – review & editing. DY: Supervision, Writing – review & editing. PR: Supervision, Writing – review & editing. DH: Conceptualization, Data curation, Investigation, Writing – original draft.
